# Ascorbic Acid for Major Depressive Disorder Treated With Modified Electroconvulsive Therapy: A Randomized Controlled Trial Protocol

**DOI:** 10.31083/AP49803

**Published:** 2026-06-25

**Authors:** Li Zhao, Yuxuan Yang, Binyang Cai, Kongyan Wu, Yuhuan Gong, Qibin Chen, Xiao Li, Jie Luo

**Affiliations:** ^1^Department of Anesthesiology, The First Affiliated Hospital of Chongqing Medical University, 400016 Chongqing, China; ^2^Department of Psychiatry, The First Affiliated Hospital of Chongqing Medical University, 400016 Chongqing, China

**Keywords:** ascorbic acid, drug-related side effects and adverse reactions, major depressive disorder, electroconvulsive therapy, treatment outcome

## Abstract

**Background:**

Major depressive disorder is marked by high prevalence and elevated suicide risk. Modified electroconvulsive therapy (MECT) is a well-established, effective, and fast-acting method for treating major depressive disorder with acute suicide control, though its application is constrained by adverse effects. Enhancing the therapeutic effectiveness and/or reducing the adverse effects of MECT holds clinical practical value. Ascorbic acid (AA) can enhance the effectiveness of some antidepressants, yet its effects on depressed patients undergoing MECT lack clinical evidence. This study investigates whether AA augments the antidepressant effects of MECT in patients with major depressive disorder.

**Methods and Analysis:**

This is a prospective, randomized, double-blind, placebo-controlled trial. We will enroll 240 patients with major depressive disorder scheduled for MECT. Participants will be treated either with intravenous AA (500 mg, n = 120) or with intravenous normal saline (n = 120), administered immediately before each MECT session, just prior to induction of general anesthesia. The primary outcome is the treatment response rate, defined as the proportion of patients with a ≥50% reduction in the 24-item Hamilton Depression Rating Scale (HAMD-24) score. Secondary outcomes include remission rate, presence of psychotic symptoms, cognition, and adverse effects. This study is expected to provide an evidence based, safe, and cost effective strategy for optimizing MECT protocols, as well as potential directions for future mechanistic investigations.

**Ethics and Dissemination:**

The study will adhere to the principles of the Declaration of Helsinki. It received approval from the Institutional Review Board of the First Affiliated Hospital of Chongqing Medical University, China (Approval No. 2024-235-01). Written informed consent will be obtained from all participants. Results will be disseminated through peer reviewed publications and conference presentations. This study will offer the first clinical evidence of the effects of AA on patients diagnosed with major depressive disorder undergoing MECT. The findings may indicate the potential of AA for refining MECT based therapeutic strategies, optimizing depression treatment protocols, and improving clinical prognosis.

**Clinical Trial Registration:**

The study has been registered on https://www.chictr.org.cn/index.html (registration number: ChiCTR2500095678; registration link: https://www.chictr.org.cn/showproj.html?proj=248072).

## Main Points

1. Modified electroconvulsive therapy (MECT) remains an irreplaceable and essential antidepressant treatment to date, despite being limited by associated adverse effects. Further exploration into methodological refinements of MECT may yield additional clinical benefits for patients with depression.

2. This study is expected to establish the initial clinical evidence for the impact of ascorbic acid in patients diagnosed with major depressive disorder undergoing MECT.

3. Whether by enhancing the therapeutic efficacy of MECT and/or mitigating its adverse effects, the findings may suggest that ascorbic acid holds potential for refining MECT-based therapeutic strategies, optimizing depression treatment protocols, and improving clinical outcomes.

## 1. Introduction

Depression is a major global mental health challenge across all age groups, as reported by the World Health Organization [1]. Major depressive disorder represents a leading cause of both mental and physical disability and ranks as a primary factor in the global disease burden [2,3]. Modified electroconvulsive therapy (MECT) is a rapid-acting and highly effective first-line intervention indicated for severe conditions of major depressive disorder, including treatment-resistant depressive episodes, and especially for the acute control of suicidal ideation [4,5]. It has been extensively incorporated into evidence-based treatment algorithms for severe major depressive disorder in contemporary clinical practice [6]. MECT under general anesthesia represents a substantial advancement over conventional electroconvulsive therapy by preventing physical injuries such as fractures, muscle strains, and bite wounds that result from generalized convulsions. However, MECT still carries adverse effects, including cognitive impairment (e.g., learning and memory deficits) and myalgia, which often elicit patient fear and poor treatment compliance [7]. Beyond modifications pertaining to the co-administration of general anesthesia and medications, improvements in the electroconvulsive parameters and conditions have been explored; the use of nonconvulsive electrotherapy and transnasal humidified rapid-insufflation ventilatory exchange during MECT is designed to enhance therapeutic effectiveness and/or mitigate adverse effects [7,8,9,10]. Further refinements in MECT treatment are likely to yield additional benefits for patients.

Ascorbic acid (AA) is an essential vitamin and a key antioxidant for humans [11]. Beyond its antioxidant properties, AA possesses supplementary advantages including a favorable safety profile, easy accessibility, high public acceptance, and good medication adherence. It has been recognized as a neuromodulator involved in many critical processes within the central nervous system [12,13]. AA is no longer regarded as a simple antioxidant, but rather an active molecule that exerts antidepressant effects through the integration of multiple pathways such as antioxidant, anti-inflammation, neurotransmitter regulation, and neurotrophic support [14,15,16,17,18,19]. The potential role of AA in mood regulation, as well as in the pathogenesis and treatment of depression, has garnered increasing attention. Evidence has suggested that AA exerts its effects through multiple pathways and targets. First, AA mitigates oxidative neuronal damage by directly neutralizing reactive oxygen species, inhibiting lipid peroxidation, and regenerating other antioxidants [14,15]. Its antidepressant effects are closely linked to modulation of glutathione synthesis and inhibition of NOD-like receptor family pyrin domain containing 3 (NLRP3) inflammasome activation, thereby reducing the maturation and release of pro-inflammatory cytokines like interleukin-1β and interleukin-18 [16]. Second, AA also serves as an essential cofactor in the biosynthesis of monoamine neurotransmitters, participating in the production of norepinephrine and neuropeptides, thus contributing to emotional regulation [16]. Third, AA modulates synaptic plasticity by regulating glutamate receptor function and influencing long-term potentiation, promotes the expression of brain-derived neurotrophic factor (BDNF), a key protein for neuronal survival and synaptic plasticity, and reverses the suppression of BDNF-related signaling pathways in animal models of depression [15,16,17,18]. Fourth, recent research has highlighted the gut–brain axis as a novel pathway for the mood-regulating effects of AA, with findings indicating that AA supplementation alters gut microbiota composition, leading to decreased serum lipopolysaccharide levels, attenuated systemic inflammation, elevated serum BDNF, and enhanced mental vitality in healthy adults [19]. Last, both preclinical and clinical evidence have supported the antidepressant potential of AA, including reversing chronic stress-induced depression-like behaviors in animal models [14,15], and enhancing the efficacy of selective serotonin reuptake inhibitors in patients with major depressive disorder [14,16].

The theoretical foundation of this study is rooted in the central role of oxidative stress in the pathogenesis of depression. A substantial body of evidence has demonstrated that major depressive disorder is closely associated with a significant imbalance between oxidant and antioxidant systems, characterized by increased generation of reactive oxygen species, elevated lipid peroxidation (e.g., increased malondialdehyde levels), oxidative damage to DNA and proteins, and dysfunction of endogenous antioxidant defense mechanisms such as superoxide dismutase [20,21]. This pathological state not only directly induces neuronal injury but also triggers neuroinflammatory responses, disrupts the metabolism and signaling of monoamine neurotransmitters, and impairs synaptic plasticity, thereby collectively constituting a fundamental pathophysiological basis of depression [20]. Notably, MECT may itself provoke transient oxidative stress responses during the induction of seizures and the administration of general anesthesia, and regulate downstream targets of synaptic plasticity in multiple brain regions [20,21,22].

AA exerts a certain degree of antidepressant effects and improves mood [23]. Literature suggests the potential involvement of oxidative stress in the pathophysiological mechanisms of depression, as evidenced by reduced plasma AA levels and elevated oxidative stress markers in patients [20,24,25]. Therefore, the mechanisms underlying the effects of MECT potentially involve the intricate participation and interplay of oxidative stress, with antioxidant interventions representing a promising therapeutic adjunct [26,27].

Research findings have indicated that AA alone has limited antidepressant efficacy but can assist in other antidepressant treatments. In preclinical studies, administration of AA ameliorated depression-like behaviors and reversed associated pathological neural alterations in mice [28,29]. Co-administration of AA with ketamine or its enantiomer (e.g., esketamine, a recently approved new rapid-acting antidepressant) significantly enhanced antidepressant efficacy, reduced psychotomimetic-like side effects, and potentially allows for lower ketamine dosing, thereby improving treatment tolerability in animal models [30,31]. Another clinical study further demonstrated that adjunctive use of AA during fluoxetine therapy for pediatric major depressive disorder resulted in a marked decrease in depressive symptoms, with no significant adverse effects observed [32]. To date, however, there have been no reported studies on the use of AA in the course of MECT.

Based on the current evidence, we hypothesize that adjunctive AA administration during MECT will enhance antidepressant effects and may even directly alleviate the adverse effects of MECT. However, direct clinical evidence derived from well-designed randomized controlled trials remains lacking. Therefore, this study will address these evidence gaps by conducting a prospective, randomized controlled trial to evaluate the clinical effectiveness of AA augmentation in individuals with major depressive disorder undergoing MECT, with a priority focus on confirming treatment response.

## 2. Materials and Methods

### 2.1 Study Design and Ethics

**Fig. 1.**
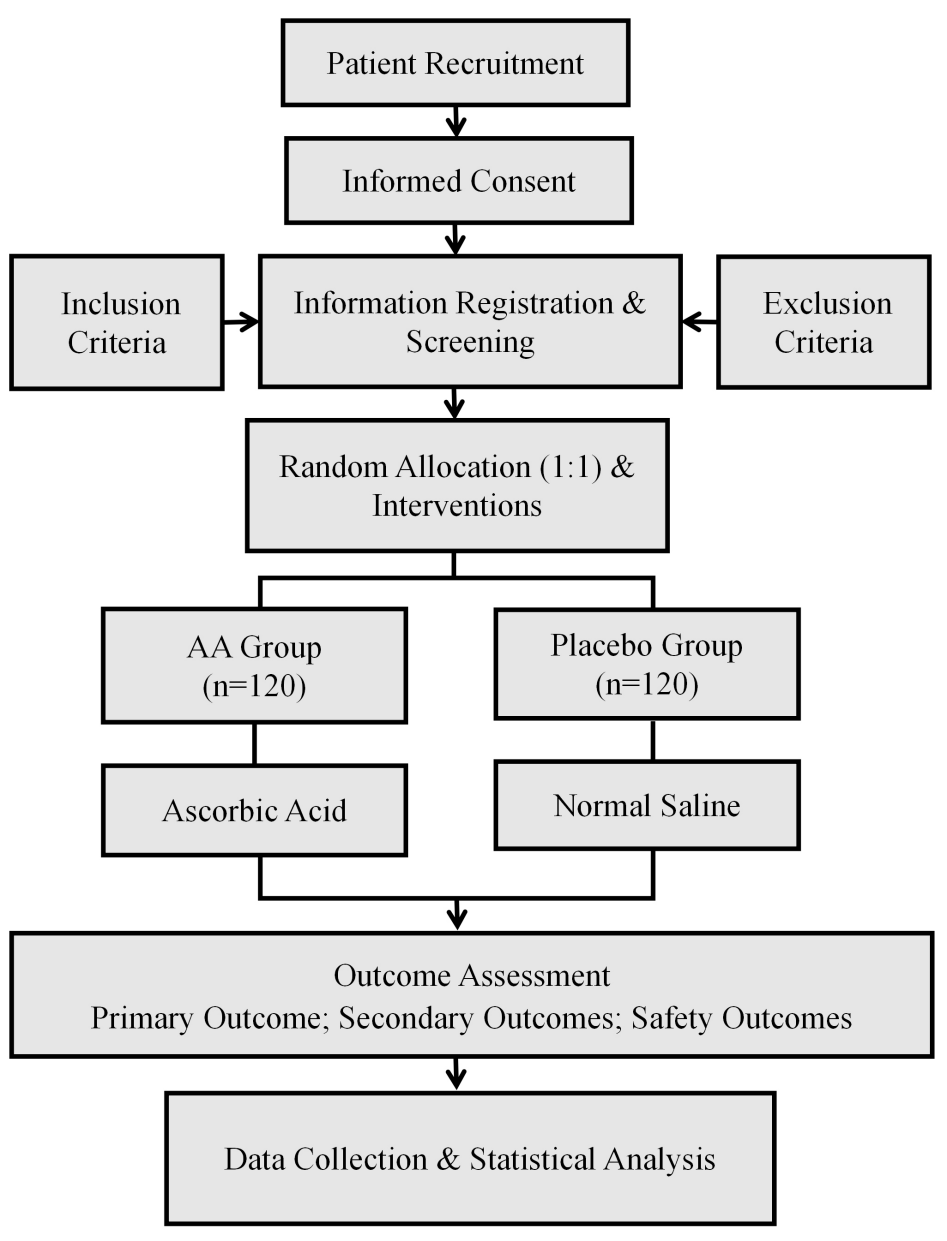
**Study design**. Abbreviations: AA, ascorbic acid.

This study is a prospective, randomized, double-blind, placebo-controlled, parallel-group clinical trial designed to assess the effect of AA treatment on the therapeutic outcomes and tolerability of MECT in patients with major depressive disorder. The protocol was drafted according to the Standard Protocol Items: Recommendations for Interventional Trials (SPIRIT), and was registered at the Chinese Clinical Trial Registry (Registration No. ChiCTR2500095678) on January 10, 2025. Fig. 1 illustrates the participant flow throughout the study.

### 2.2 Participants

Potential participants will be screened at the Department of Psychiatry in the First Affiliated Hospital of Chongqing Medical University. Eligible participants will be enrolled after providing written informed consent and may withdraw from the study at any point.

Inclusion Criteria: (1) Diagnostic and Statistical Manual of Mental Disorders, Fifth Edition (DSM-5) diagnosis of major depressive disorder requiring MECT and hospitalization; (2) aged 18–59 years; (3) body mass index (BMI) between 18.5–30 kg/m^2^; (4) physical status (American Society of Anesthesiologists, ASA) I–II, without major comorbidities; (5) willingness of the patients and/or their legal guardian to offer written informed consent.

Exclusion Criteria: (1) comorbid with severe medical and/or surgical diseases, including significant cardiovascular or cerebrovascular diseases, uncontrolled hypertension, elevated intracranial pressure, or the presence of cardiac pacemakers or intracranial implants; (2) co-occurring psychiatric disorders (e.g., schizophrenia); (3) conditions that may interfere with efficacy assessments (e.g., catatonia (diagnosed by experienced psychiatrists according to DSM-5 criteria), cognitive impairment, illiteracy, or intellectual disability); (4) contraindications to anesthesia or MECT (e.g., full stomach, severe pulmonary dysfunction, known allergy to anesthetics); (5) known allergy to ascorbic acid or any contraindications to its administration; (6) history of alcohol or other substance dependence; (7) previous ineffectiveness or serious adverse effects of MECT; (8) pregnancy; (9) participation in another clinical trial within the past year.

Participants will be discontinued from the study if any of the following occur: (1) serious adverse effect necessitating termination of the intervention; (2) request by the patient and/or family to withdraw; (3) clinical circumstances evaluated by the treating psychiatrist that warrant discontinuation of MECT (e.g., remission according to the 24-item Hamilton Depression Rating Scale (HAMD-24) criteria, defined as a score of <10 on two consecutive assessments, occurrence of severe cognitive impairment, intolerable reactions, or no clinical improvement after eight MECT sessions, etc.); (4) violation of trial protocol.

### 2.3 Randomization

We plan to enroll 240 participants. An independent investigator, who is not otherwise involved in the trial, will generate a computer-based randomization sequence. Participants will be randomized using a stratified block randomization method, with stratification based on age, sex, and baseline 24-item Hamilton Depression Rating Scale (HAMD-24) score. A fixed block size of 4 will be used. Subjects will be assigned 1:1 (n = 120 per group) to either the ascorbic acid group (AA Group) or the placebo group (Placebo Group). Concealment of allocation will be ensured until intervention using sequentially numbered, opaque, sealed envelopes. All personnel will be blind to the treatment of the subjects throughout the trial. The research staff responsible for participant enrollment will safeguard the allocation sequence but will have no involvement in the randomization process, clinical interventions, or outcome assessments. All treating clinicians (psychiatrists, anesthesiologists, nurses), outcome assessors, participants, and their families will remain blind to group assignments during the duration of the trial. Emergency unblinding is permitted only for serious adverse events requiring intervention knowledge for clinical management, per the predefined procedure.

### 2.4 Intervention

Participants will receive an intravenous infusion of AA or the matching placebo (saline) immediately prior to anesthesia induction for each MECT session. This administration is fully synchronized with the MECT procedure, meaning that each intervention (or placebo) dose is given before every electroconvulsive therapy session. Participants in the AA Group will receive 500 mg of AA (2 mL containing 0.5 g; Xinhua Pharmaceutical Co., Ltd., Zibo, Shandong, China),constituted by diluting the solution with normal saline to a final volume of 100 mL. Participants in the Placebo Group will receive 100 mL of normal saline alone. All study solutions will be prepared under aseptic conditions by independent personnel not involved in the trial’s clinical conduct. The final solutions for both groups are visually indistinguishable (clear and colorless liquids) and will be dispensed in identical, tamper-evident, light-proof containers to ensure blinding throughout preparation, handling, and administration.

### 2.5 MECT Procedure

The MECT protocol is determined by the treating psychiatrist, initiated with up to four weekly sessions during the first treatment week before transitioning to a maintenance schedule of three sessions per week. The procedure utilizes a Thymatron DGx device (Somatics, LLC, Lake Bluff, IL, USA) with bitemporal electrode placement positioned 5 cm superior to the lateral orbital rim. Stimulation parameters consist of square-wave stimulation at 900 mA, with 125 bidirectional pulses per second and a brief-pulse width of 1.5 ms [33]. The initial electrical charge is calculated using the half-age method, with subsequent doses adjusted in 5% increments per session based on continuous electroencephalography (EEG) monitoring of seizure characteristics including morphology and duration. Anesthetic administration includes intravenous atropine (0.5 mg), propofol (2 mg/kg), and succinylcholine (1 mg/kg), with dosage customization according to individual patient response to achieve optimal seizure expression. The electrical stimulus will be delivered immediately following the cessation of succinylcholine-induced fasciculations, under conditions of adequate oxygenation. Treatment efficacy is confirmed through electrographic seizure duration exceeding 25 seconds [34,35] and characteristic EEG patterns demonstrating polyspike-wave complexes, 3-Hz spike-and-wave discharges, and postictal suppression [33,34,36].

### 2.6 Outcomes

Outcome categories and their assessment timepoints are detailed in Table 1. Data will be collected by independent investigators who are blind to group allocation and intervention assignments.

**Table 1.** **Outcome measurements**.

Outcomes	Measurements	Scheduled time		
		Baseline	Each MECT	Before discharge
Primary outcome:				
Treatment response rate	HAMD-24 scale	×		×
Secondary outcomes:				
Depression remission rate	HAMD-24 scale	×		×
Suicide ideation remission rate	HAMD-24 scale (item 3)	×		×
Associated psychotic symptoms	BPRS scale	×		×
Cognition	MoCA scale	×		×
Electroconvulsive parameters	Field recording		×	
Anesthesia related data	Field recording		×	
Blood biomarkers	Blood detection	×		×
Safety outcomes:				
Adverse effects of ascorbic acid	Observation and inquiry		×	×
Adverse effects of MECT	Observation and inquiry		×	×

BPRS, Brief Psychiatric Rating Scale; HAMD-24, 224-item Hamilton Depression Rating Scale; MECT, modified electroconvulsive therapy; MoCA, Montreal Cognitive Assessment; "×" symbol indicates the time point at which each assessment is carried out.

For the confirmatory index, the primary outcome is the treatment response rate of the group. Response is operationally defined as achieving at least a 50% reduction from baseline on the HAMD-24 total score by the end of treatment [37]. A qualified psychiatrist rates the 24 items of the HAMD-24 on a 0–4 severity scale (0 = none, 1 = mild, 2 = moderate, 3 = severe, 4 = very severe) of 24 items [37]. Treatment response rate is expressed as the percentage of patients responding to MECT relative to the total number of patients in each group. The rationale for selecting the “response rate” as the primary outcome is as follows: (1) the response to an antidepressant treatment with a ≥50% reduction in the scale score has been generally recognized as a clinically meaningful treatment success intuitively reflecting the efficacy intensity of the intervention measures in confirmatory studies [38,39]; and (2) is an internationally accepted consensus standard for antidepressant efficacy evaluation by the US Food and Drug Administration (FDA) and European Medicines Agency (EMA) [40,41], and has been widely used as the primary outcome in evaluating the efficacy of MECT as well [42,43].

Secondary outcomes are as follows. The rate of remission of depression after MECT treatment is assessed with the HAMD-24 scale. Remission requires HAMD-24 score <10 after two sequential sessions, and the rate is derived from the number of remitters divided by the total participants per group [44]. For suicidal ideation (SI), defined as a score ≥2 on HAMD-24 item 3, the remission rate represents the proportion of baseline SI-positive patients achieving a score <2 after MECT [45]. Assessment of accompanying psychotic symptoms will be done with the 18-item Brief Psychiatric Rating Scale (BPRS), which uses a 7-point scale per item. The total score (18 to 126) serves as an indicator of severity; higher values indicate worse symptoms [46]. The Montreal Cognitive Assessment (MoCA) serves to rapidly assess cognitive function, providing a total score of 30; better function is indicated by higher scores [47]. Parameters recorded post-stimulation include: 3 Hz spike and wave activity; multiple spike activity; post-seizure suppression; and seizure duration. Based on common practice in MECT trials, a seizure with a duration longer than 25 s is considered indicative of an effective treatment [33,34], and seizure quality on EEG will be assessed with a specific standardized scale. Vital signs (oxygen saturation, heart rate, blood pressure, etc.), and anesthetic indices (duration, dosages of anesthetics) during each MECT treatment will be recorded. Blood samples will be collected to assess potential mechanistic biomarkers including AA, interleukin-1β (IL-1β), IL-6, tumor necrosis factor alpha (TNF-α), and BDNF.

Safety outcomes will be monitored as well, focusing on adverse events (AEs) associated with AA and/or MECT. An AE constitutes any unforeseen medical occurrence after the intervention. Although AA is generally well-tolerated, any newly observed side effects will be recorded, as well as those of MECT, including memory deficits and muscle pain. Participants will be instructed to report any AEs, which will be assessed by a physician and documented. The occurrence of a serious AE will trigger unblinding, administration of necessary treatment, and mandatory reporting to the Institutional Review Board.

### 2.7 Sample-Size Estimation

The primary outcome is the response rate of the group to MECT. As no prior data exist regarding the efficacy of AA augmentation in individuals diagnosed with major depressive disorder receiving MECT, the required sample size was determined based on our pilot study (*n *= 40, i.e., 20/gp) conducted over 6 mo, which indicated a response rate of 60% (12/20) in the placebo group and an improvement of 20% (16/20) in the AA group. With a two-sided α of 0.05, a power of 85% (1-β = 0.85), and 1:1 allocation, the calculation performed with PASS software (version 2021, NCSS, LLC, Kaysville, UT, USA) indicated a requirement of 93 participants/gp. To allow for an anticipated 22.5% attrition rate, a target enrollment of 120 participants/gp is planned, resulting in a final total sample of 240 subjects.

### 2.8 Statistical Analysis

The intention-to-treat (ITT) analysis set in this study will include all randomly assigned participants, regardless of whether they received MECT treatment or completed the follow-up assessments. Analyses will be conducted using SPSS (version 26, IBM Corp., Armonk, NY, USA). Missing outcome or covariate data, including those from participants in the ITT set without post-baseline data, will be handled using multiple imputation, and a mixed model for repeated measures (MMRM) will be used where appropriate. For the primary outcome (the rate of response to MECT), binary logistic regression, with treatment group as the independent variable and pre-specified stratification factors included as covariates, will serve as the primary analytical method, and results will include an adjusted odds ratio (aOR) with a 95% confidence interval. Additionally, potential interaction effects between the stratification factors (age, sex, baseline HAMD-24 score) and the treatment group will be explored within this model for the primary outcome. The Chi-square test will be used as a supplementary analysis. Categorical variables, expressed as counts (percentages), will be compared between groups using the Chi-square test (or the Fisher’s exact test). Continuous variables will be presented as mean (standard deviation) (if normally distributed) or median (interquartile range) (if non-normally distributed), and compared using *t*-test or nonparametric tests (e.g., Mann-Whitney U test), as appropriate. For secondary-outcome analyses, logistic regression will be performed to adjust for potential confounders. For multiple comparisons, the Bonferroni correction will be applied for the secondary and exploratory outcomes. Specifically, the Bonferroni correction will be applied uniformly across all pre-specified secondary outcomes to control the family-wise error rate. We acknowledge that this is a conservative approach, which prioritizes the control of Type I error over statistical power for these outcomes. Given their exploratory feature, between-group differences in blood biomarker levels from baseline to post-treatment will be compared using an analysis of covariance (ANCOVA) adjusted for baseline values, and the Benjamini–Hochberg false discovery rate (FDR) procedure will be applied to correct for multiple testing. Pre-specified subgroup analyses will be conducted based on age (<45 and ≥45 years), sex (male and female), and baseline HAMD-24 score (divided at the median). These analyses will be performed by including an interaction term between the treatment group and the subgroup variable in the primary statistical model (binary logistic regression). Given the exploratory and hypothesis-generating nature of these subgroup analyses, no multiplicity adjustment will be applied to the interaction tests, and the results will be interpreted with caution. Sensitivity analyses will include the per-protocol (PP) analysis and complete-case analysis, an evaluation using alternative missing-data-handling strategies, and re-analyses excluding specific populations (e.g., protocol violators) to assess the robustness of the findings. All hypothesis tests will be two-tailed, and a statistical significance level will be set at* p *< 0.05.

## 3. Discussion

This protocol is for a randomized, controlled trial primarily investigating the potential enhancement of antidepressant efficacy of MECT for major depressive disorder by AA treatment. Additionally, secondary outcomes in exploratory studies will include evaluating the effects of AA on other variables of MECT and related general anesthesia, and whether AA can prevent worsening of, or even improve, the adverse effects. We will also explore the associated molecular biomarkers potentially underlying these effects. To the best of our understanding, this represents the first randomized controlled trial designed to evaluate the effects of AA treatment on individuals diagnosed with major depressive disorder undergoing MECT, specifically targeting the potential augmentation of AA treatment on MECT efficacy.

This study will reasonably and rigorously provide robust clinical evidence and will adhere to the Consolidated Standards of Reporting Trials (CONSORT) 2025 Statement guidelines [48]. The prospective, randomized, blind, placebo-controlled method, along with a justified sample-size estimation, allows for the potential detection of intergroup differences and the identification of relevant effects through comparative analysis. Patient selection will be restricted to individuals with major depressive disorder who were clinically indicated for MECT. Both the electroconvulsive therapy and anesthesia procedures will be administered according to standard clinical routine protocols, unaffected by trial participation, thereby ensuring balanced implementation across study groups.

The proposed dose of AA has been determined based on the safety range specified in the manufacturer’s prescribing information and previously reported doses in the literature, optimizing therapeutic efficacy and ensuring medication safety [32,49]. The dose of AA was selected based on a comprehensive consideration of clinical evidence, pharmacokinetic principles, and the safety profile. Clinical studies have demonstrated that adjunctive use of AA (500 mg twice daily) significantly improved depressive symptoms and was well-tolerated in both adult and pediatric patients with major depressive disorder [32,50]. Pharmacokinetic studies have indicated that a 500 mg dose maintained plasma AA concentrations above the neuroprotective threshold (>50 μmol/L), while avoiding gastrointestinal adverse effects and absorption saturation associated with single doses exceeding 1000 mg [51]. Furthermore, high-dose intravenous AA (median 22.5 g/d, up to 100 g/d) has not been associated with a higher risk of adverse events than placebo in randomized controlled trials [52], further supporting the rationale and safety of the 500 mg intravenous dose to be used in this study. Nevertheless, all potential adverse events will be closely monitored and systematically documented, with appropriate interventions provided as necessary, thereby ensuring the safety and well-being of participants throughout the study.

Comprehensive monitoring of outcomes captures intervention efficacy from multiple angles: clinical endpoints including depressive symptoms; suicidality; cognitive function; psychotic symptoms; seizure parameters; anesthetic variables; and blood biomarkers. This multidimensional approach is designed to provide both efficacy evidence and possible mechanistic clues [53,54,55,56]. Measurements will be collected by independent investigators who remain blind to both the experimental intervention and group allocation. In addition, all subjective rating scales used are currently recognized and widely used standardized instruments for the relevant measurements. To control the measurement quality and enhance the reliability of the results, assessments will be conducted by psychiatrists who have received strict, study-specific training.

The mechanistic rationale for this study focuses specifically on pathways potentially relevant to AA and MECT synergy. AA may target mechanisms that complement MECT, primarily through its potent antioxidant and anti-inflammatory effects [14,15,16], which could mitigate oxidative stress associated with both major depressive disorder and the MECT procedure itself. Furthermore, AA’s role in modulating BDNF and synaptic plasticity [15,16,17,18] represents another key pathway, as BDNF signaling is implicated in the therapeutic action of MECT. The biomarker analyses in this study will preliminarily explore these specific mechanisms, antioxidant/anti-inflammatory effects and BDNF modulation—to provide clues for future research.

Therefore, we hypothesize that the adjunctive use of the potent antioxidant AA during MECT may directly counteract oxidative stress processes and affect potential downstream targets. By mitigating cumulative oxidative neuronal damage, we propose that AA creates a more favorable condition for the antidepressant effects of MECT, representing a potentially key mechanistic pathway through which AA enhances the therapeutic efficacy of MECT. Although further mechanistic investigation is warranted, this study will provide clinical evidence that may support translational applications.

Some limitations of this study may exist. First, the single-center design may limit generalizability, though standardized protocols maximize internal validity. Second, despite rigorous rater training, a degree of subjectivity inherent to clinician-rated scales remains unavoidable. Third, regarding the electrophysiological monitoring of MECT sessions, the EEG-based evaluation of seizure adequacy relied on traditional observational methods (e.g., visual inspection of seizure duration and morphology). This approach is inherently subjective and lacks the objective, quantitative metrics that modern automated EEG analysis tools could provide, potentially introducing assessment bias. Fourth, the biomarker analyses are exploratory in nature. While they may reveal valuable associations, the correlations observed cannot establish causality between the changes in biomarker levels and the clinical outcomes.

## 4. Summary

This proposal is for a double-blind, randomized controlled trial to assess the efficacy, safety, and potential correlated biomarkers of AA as an augmentation strategy for MECT among individuals diagnosed with major depressive disorder. This study is expected to provide an evidence-based, safe, and cost-effective strategy for optimizing MECT protocols, and also offer potential directions for future mechanistic investigations.

## Data Availability

Subsequent data supporting future derived findings once published, can be available from the corresponding author upon reasonable request.
